# Predictors of accessing seasonal malaria chemoprevention medicines through non-door-to-door distribution in Nigeria

**DOI:** 10.1186/s12936-024-04964-5

**Published:** 2024-05-03

**Authors:** Sikai Huang, Kevin Baker, Taiwo Ibinaiye, Olusola Oresanya, Chuks Nnaji, Sol Richardson

**Affiliations:** 1 Vanke School of Public Health, Tsinghua University, Beijing, 100083 China; 2 Malaria Consortium UK, The Green House, 244-254 Cambridge Heath Road, London, E2 9DA UK; 3 Department of Global Public Health, Karolinska Institute, Stockholm, Sweden; 4 Malaria Consortium Nigeria, 33 Pope John Paul Street, Maitama, Abuja-FCT Nigeria

**Keywords:** Seasonal malaria chemoprevention, Nigeria, Door-to-door delivery, Adherence, Adverse reaction

## Abstract

**Background:**

In Nigeria, seasonal malaria chemoprevention (SMC) is typically administered door-to-door to children under five by community medicine distributors during high transmission seasons. While door-to-door distribution (DDD) is exclusively employed in Nigeria as part of standard operating procedures of SMC programmes, some households access SMC through non-DDD channels, such as fixed-point distributions, health facilities, and private purchase. However, analysis of access to SMC medicines through non-DDD has been limited, with little evidence of its outcomes on adherence to the three-day complete course of SMC medicines and caregiver actions in the event of adverse reactions to SMC medicines.

**Methods:**

Data were obtained from SMC end-of-round coverage surveys conducted in Nigeria in 2021 and 2022, including 25,278 households for the analysis. The proportion of households accessing SMC medicine through non-DDD and the distribution of various non-DDD sources of SMC medicines were described. Multivariate random-effects logistic regression models were performed to identify predictors of accessing SMC medicines through non-DDD. The associations between non-DDD, and caregiver-reporting of adherence to complete administration of SMC medicines and caregiver actions in the event of adverse reactions to SMC medicines were also assessed.

**Results:**

Less than 2% (314/24003) of households accessed SMC medicines through non-DDD in the states surveyed. Over 60% of non-DDD access was via health facility personnel and community medicine distributors from different locations. Variables associated with non-DDD access included heads of household being born in the local state (OR = 0.68, 95% CI 0.47 to 0.90), households residing in the study state since the first cycle of the SMC round (OR = 0.39, 95% CI 0.17 to 0.88), households with high wealth index (OR = 1.36, 95% CI 1.01 to 1.82), and caregivers hearing about date of SMC delivery in the previous cycle (OR = 0.18, 95%CI 0.14 to 0.24). Furthermore, non-DDD was associated with reduced SMC adherence and higher caregiver non-reporting of adverse reactions to SMC medicines in children compared with DDD.

**Conclusion:**

This study provides evidence on the characteristics of households accessing SMC medicines through non-DDD and its potential negative outcomes on adherence to SMC medicine and adverse reaction reporting, underscoring potential implementation issues that may arise if non-DDD delivery models are adopted in SMC, particularly in places where DDD had been firstly used.

**Supplementary Information:**

The online version contains supplementary material available at 10.1186/s12936-024-04964-5.

## Background

In 2021, African countries accounted for approximately 95% of global malaria cases and 96% of malaria deaths, with children under the age of five constituting about 80% of all malaria-related deaths in Africa [1, 2]. Nigeria bears the highest burden of malaria globally, with 38.4% of global malaria deaths in under-five children in 2021 [2]. Since 2012, the World Health Organization (WHO) has recommended seasonal malaria chemoprevention (SMC) to address malaria burden in children aged 3–59 months, which has been shown to be highly effective in preventing morbidity and mortality from malaria [3, 4]. In 2022, SMC was implemented in 15 sub-Saharan African countries with marked seasonality in malaria transmission, reaching an estimated 45 million eligible children [5–7]. In Nigeria, a total of 21 eligible states with seasonal malaria transmission have been reached by SMC, targeting a population of 27.1 million eligible children in 2022 [8].

SMC involves administration of courses of anti-malarial sulfadoxine-pyrimethamine (SP) and amodiaquine (AQ) to children aged 3–⁠59 months to prevent malaria by maintaining therapeutic drug concentrations in the blood during the high transmission season [9]. Administration is supervised by SMC community distributors (CDs), who provide one dose of SP and AQ in person on the first day (Day 1) [6]. Following Day 1 SPAQ, caregivers administer a daily dose of AQ unsupervised on the second (Day 2) and third days (Day 3). This three-day complete course and subsequent 28-day protective period are referred to as a “cycle” of SMC. In Nigeria, annual SMC rounds comprise four or five monthly cycles depending on the state and length of the transmission season [8].

Door-to-door distribution (DDD), which involves household visits by CDs, has been successfully used in distribution of HIV self-testing kits, child immunization campaigns, mass drug distribution for neglected tropical diseases, and SMC [10–12]. In Nigeria, a hybrid distribution approach, fixed-point distribution and DDD, was employed when SMC firstly introduced in Nigeria; however, DDD has been exclusively approved in SMC programmes as part of standard operating procedures since 2016 [7]. Some households may access SMC medicines through non-DDD (i.e., obtain medicines outside of household visits by CDs), which is not affiliated with SMC programmes [6]. Non-DDD includes gathering children at fixed points by CDs, medicine distribution by health workers at an outpatient department or outreach clinic, and distribution of medicines at makeshift locations such as workplaces through CDs; these may be employed to complement DDD to ensure higher coverage based on the actual on-site circumstances [6]. Informal non-DDD access may also occur, such as unofficial fixed-point distribution in markets, family or friends, or private purchase of medicines that should be provided for free through SMC programmes.

DDD is preferable over official fixed-point distribution and other methods to facilitate more equitable access and higher coverage, with caregivers advocating DDD due to easy accessibility and active engagement with CDs [13–16]. However, some households still access SMC through non-DDD. SMC coverage and implementation research conducted by Malaria Consortium demonstrated that the proportions of households receiving SMC treatment through non-DDD in six African countries were below 2.5% in 2021 and below 1.5% in 2022 [17, 18]. These proportions are small but non-negligible considering the scale of DDD for SMC. Furthermore, the latest WHO guidelines, which give greater flexibility to implementers in tailoring delivery approaches, may lead to a significant increase in the proportion of non-DDD [6, 7]. However, the characteristics of recipients accessing SMC through non-DDD, the factors predictive of their use of access method, and the outcomes of non-DDD administration of SMC have yet to be described.

Previous studies investigated sociodemographic factors associated with non-DDD receipt of preventive medicines in other interventions. One study conducted in Uganda on community-directed mass drug administration for schistosomiasis and hookworm infections found that household-level factors such as low quality of housing construction and household head being of village majority ethnicity was associated with non-receipt of other preventive medicines [19]. Another study in thirteen sub-Saharan African countries based on mixed distribution (DDD and fixed-point plus outreach) of Vitamin A supplementation to children reported that caregivers with formal schooling were more likely to access supplements for their children at fixed sites plus outreach locations but less likely through DDD [20]. Two qualitative studies conducted in sub-Saharan Africa found that a lack of understanding of the hazard of malaria infection and the purpose of health campaigns and mistrust in effectiveness and safety of free preventive medicines were barriers to DDD; however, their comparison groups were not recipients of non-DDD [16, 21]. No quantitative study to date has investigated predictors of access to SMC medicines through non-DDD, compared with DDD.

**Fig. 1:**
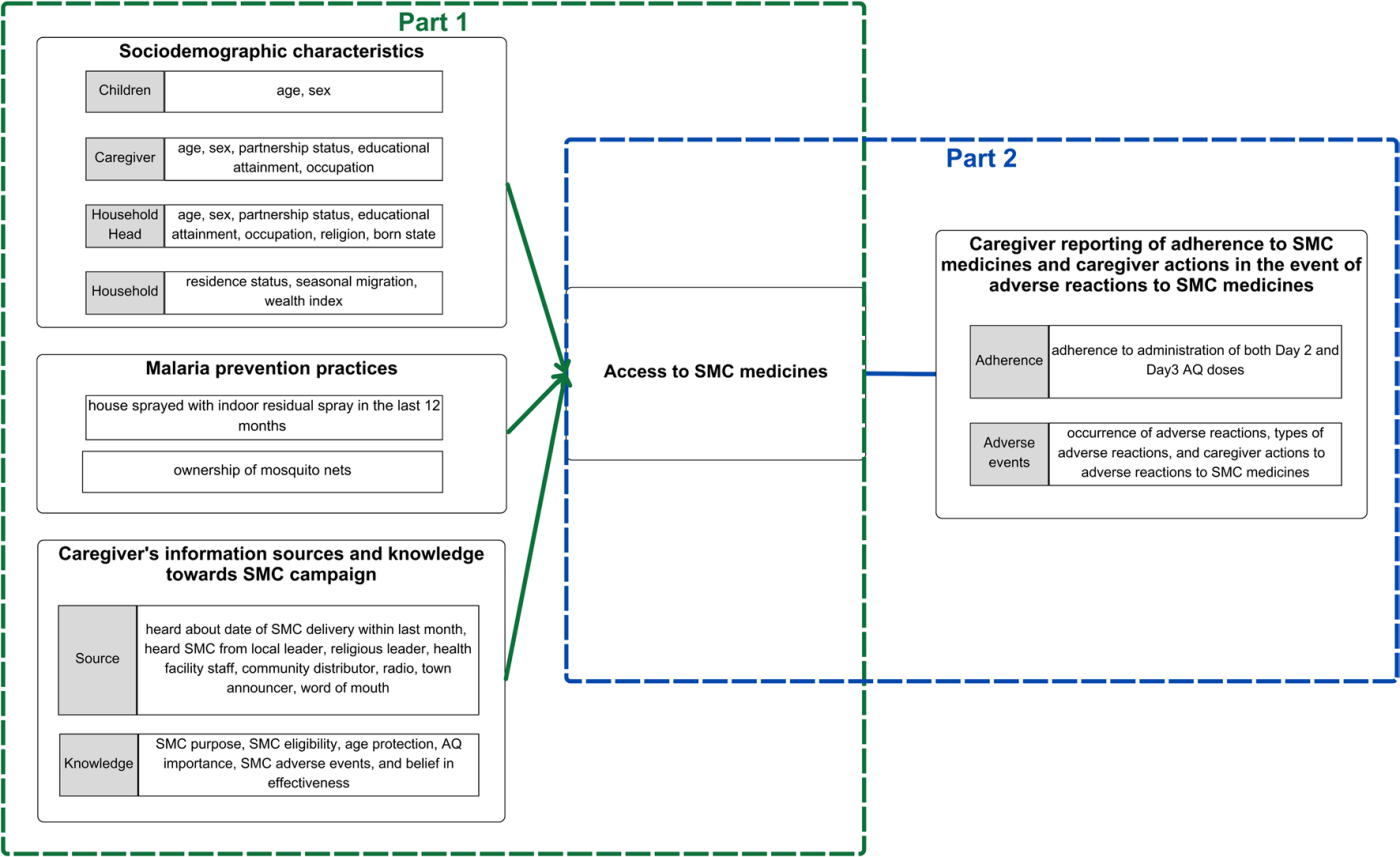
Study framework. Part 1, to identify predictors of accessing SMC medicines through non-DDD; Part 2, to assess associations between distribution approaches and caregiver reporting of adherence to SMC medicines and caregiver actions in the event of adverse reactions to SMC medicines. *SMC* seasonal malaria chemoprevention, *AQ* amodiaquine

More knowledge of distribution approaches accessed by recipients to access SMC medicines is crucial. It can provide new insights into identifying whether noncompliance with field protocols by implementers, caregiver self-decision, or both play a role in accessing SMC through non-DDD channels. This knowledge can help stakeholders, for example, to develop strategies to mitigate against potential lower adherence to SMC medicines from the use of non-DDD. Therefore, we aimed to characterise recipients of SMC through non-DDD methods in Nigeria using data from annual SMC End-of-Round (EoR) coverage surveys commissioned by Malaria Consortium. This study focused on Nigeria due to its relatively higher incidence of households accessing SMC through non-DDD and the availability of a large sample size. The primary study objectives were to (i) estimate SMC coverage by non-DDD; (ii) describe the characteristics of households accessing SMC medicines through non-DDD; and (iii) identify predictors of accessing SMC medicines through non-DDD (Fig. 1, part 1). Additionally, it could be hypothesized that non-DDD might lead to lower adherence to SMC medicines and differences in caregiver responses to adverse reactions. Therefore, another objective was to assess associations between non-DDD access to SMC medicines and caregivers’ self-reported adherence to administration of SMC medicines and caregivers’ self-reported responses to adverse reactions (Fig. 1, part 2).

## Methods

### Data source

Data were obtained from EoR surveys conducted after the final cycle (either the fourth or fifth cycle depending on the state) of each annual SMC round in 2021 and 2022. These surveys allowed measurement of coverage, implementation quality, and SMC impact on malaria prevention through state-representative samples. Multi-stage random sampling was employed to select households in the study states with equal target sample size by state regardless of population. More detailed information on the EoR survey protocol is described elsewhere [18, 22]. EoR surveys employed computer-assisted personal interviews using SurveyCTO. In each household a roster of all children aged 3–⁠59 months at the start of the SMC round was taken; SurveyCTO randomly selected one eligible child, and all survey questions related to that child, their caregiver, and household. Informed consent was obtained from all respondents prior to the interview. All data were anonymized.

### Study area and population

**Fig. 2:**
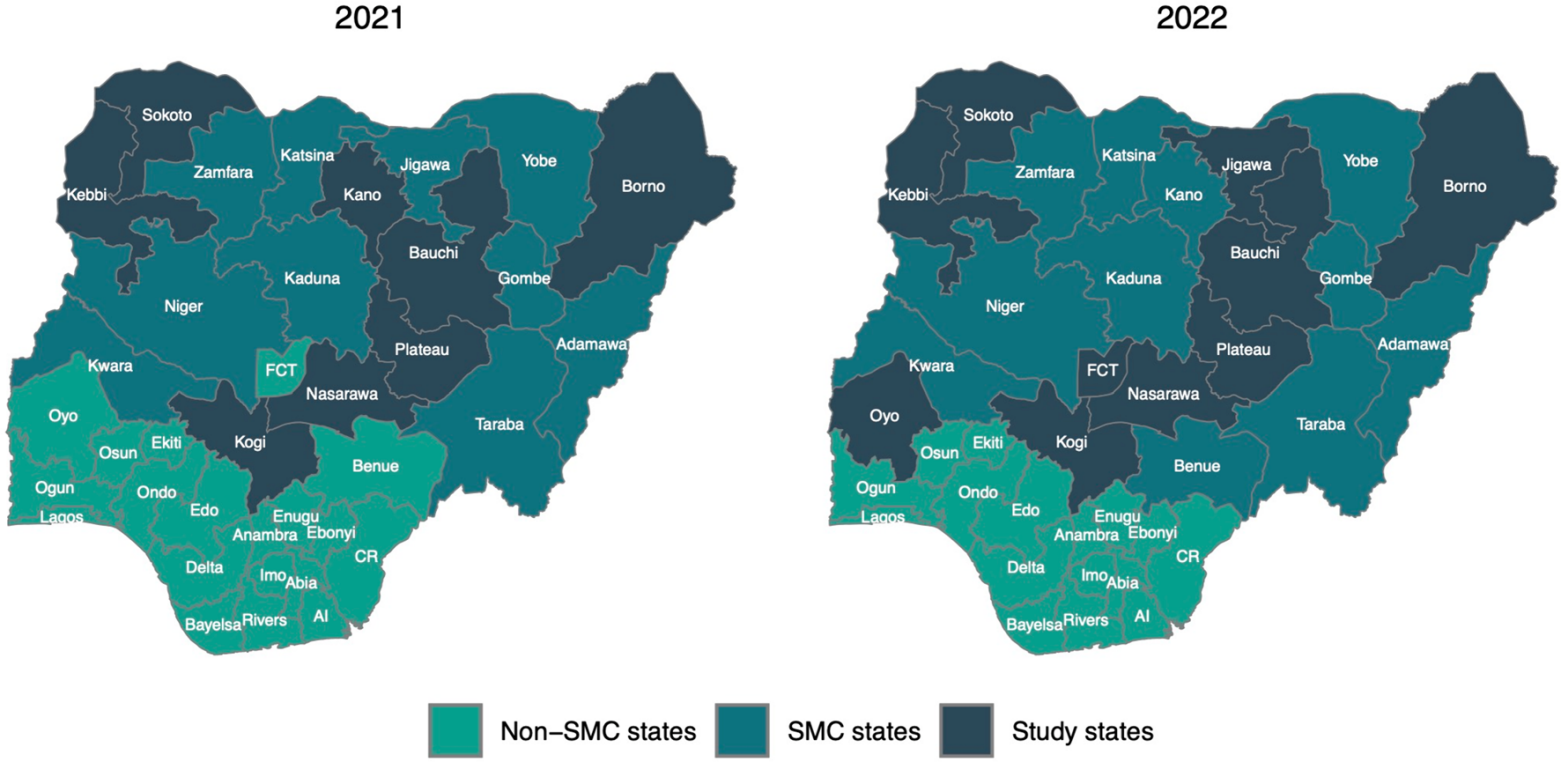
SMC settings in Nigeria in the two annual rounds in 2021 and 2022. *AI* Akwa Ibom, *CR* Cross River, *FCT* Federal Capital Territory

**Fig. 3:**
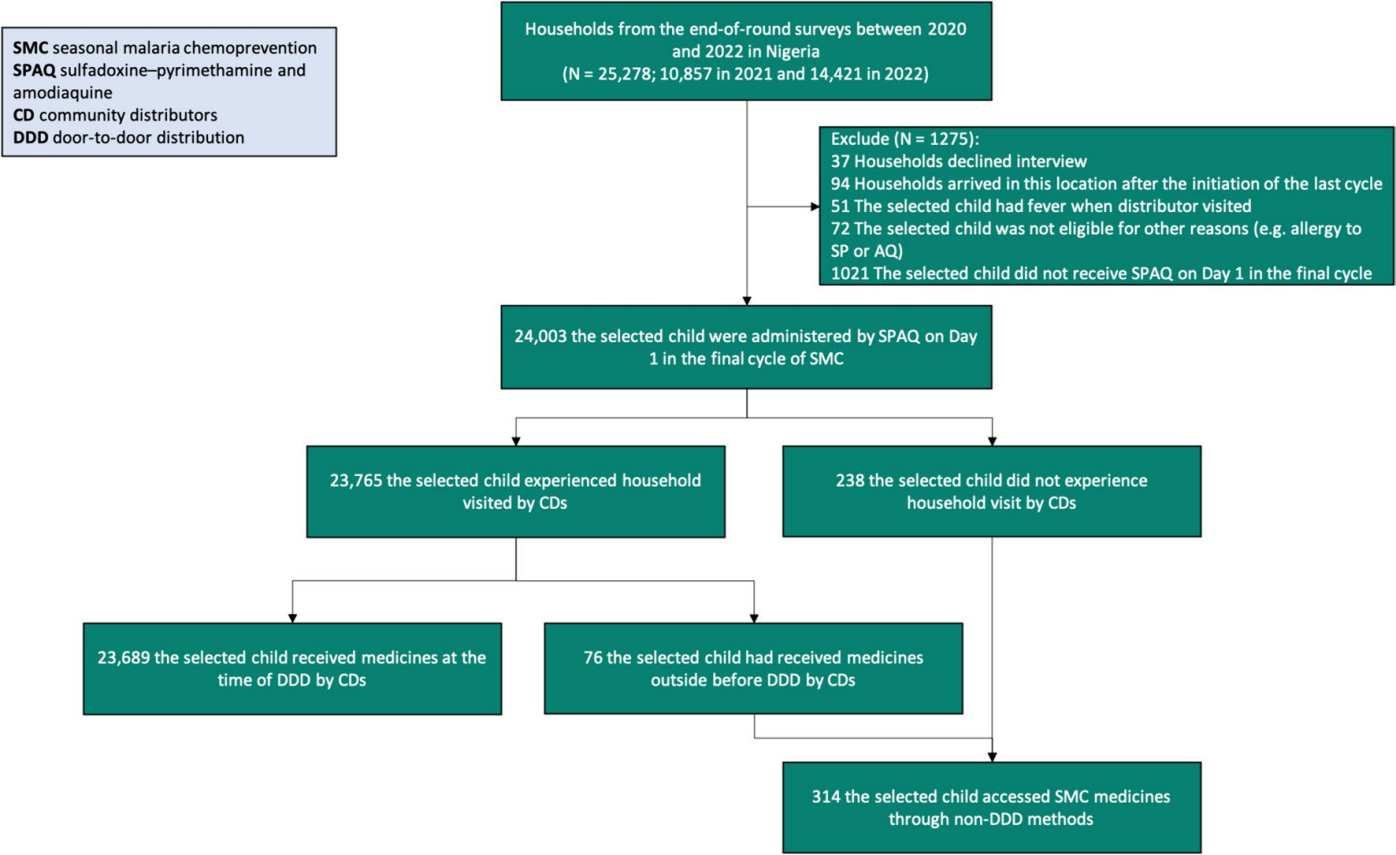
Study sample flow diagram for analysis of access to SMC medicines in Nigeria in 2021 and 2022

This study focused on eight states in 2021 and ten states in 2022 with SMC and EoR surveys (Fig. 2). The analytic sample comprised 25,278 households. Households were excluded if they declined the interview, they arrived in the locations with SMC implementation after the initiation of the final cycle, or their children had a fever during household visits by CDs or were ineligible for other reasons (e.g., allergy to SP or AQ). Households were further excluded in which children did not receive Day 1 SPAQ in the final cycle of each annual round in 2021 and 2022. The final sample included 24,003 households. Figure 3 presents the sample flow diagram.

### Variables

**Fig. 4:**
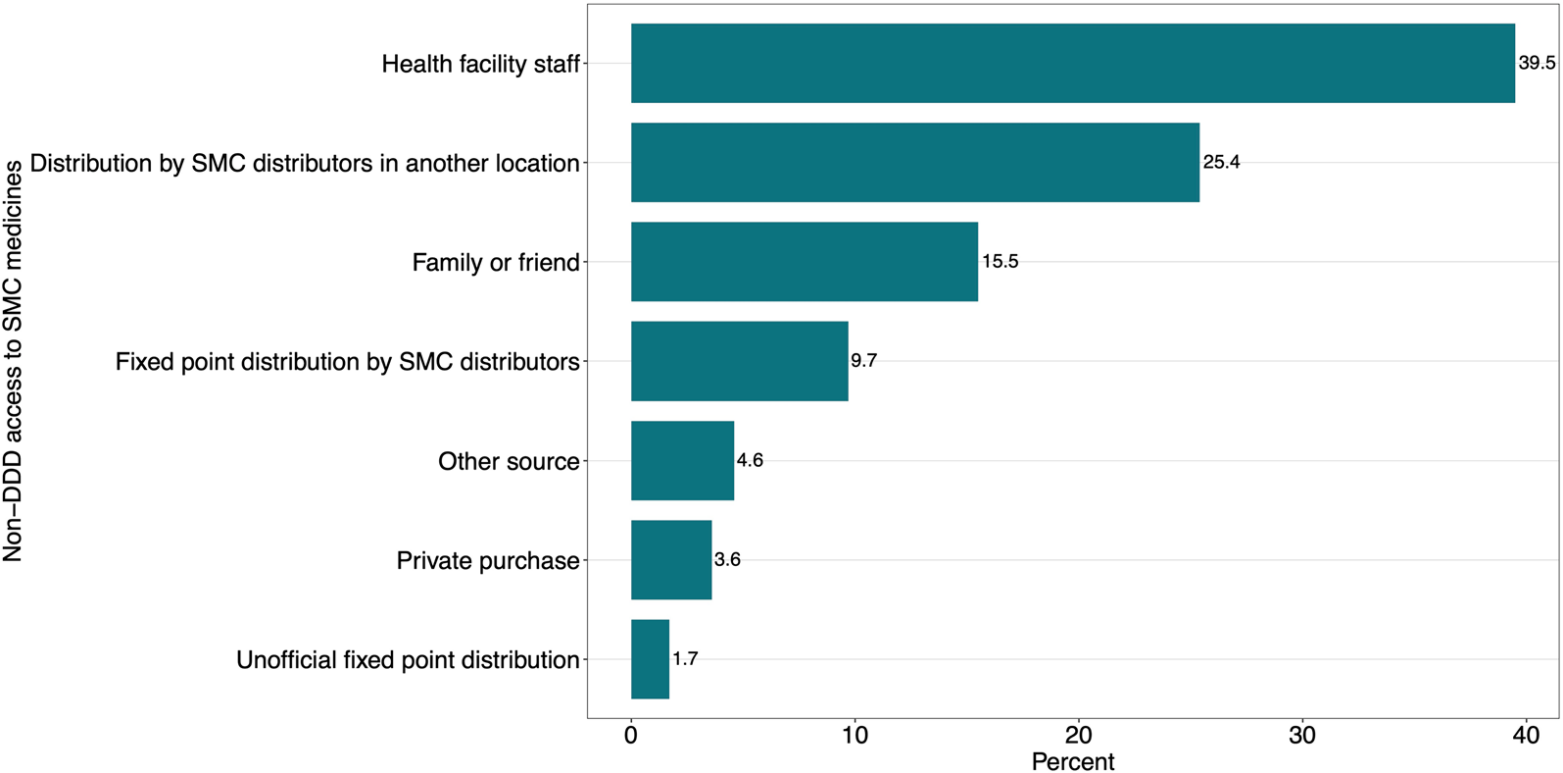
Distribution of non-DDD access to SMC medicines (N = 314). Percent were calculated by weighted sample. *SMC* seasonal malaria chemoprevention

The outcome of interest was defined as whether Day 1 SPAQ administered to an eligible child was obtained by either DDD by CDs or non-DDD. Non-DDD access to SMC medicines refers to the situation where the medicines administered by caregivers to eligible children came from a source other than household visits by CDs, regardless of whether the CDs visited their household or not (see Fig. 4 for a list of all non-DDD sources). Households that were approached and provided medicines by the CDs but had already administered medicines to their children before the visit were also classified as non-DDD.

Three groups of variables were considered potential predictors of non-DDD access to SMC medicines through non-DDD. These groups included (i) sociodemographic characteristics of children, caregivers, the head of household, and household, (ii) malaria prevention practices, and (iii) caregivers’ information sources and knowledge of SMC. Table 1 provides a summary of these variables.

**Table 1:** Sociodemographic characteristics of children, caregivers, household of heads, and households in the context of SMC in Nigeria in 2021 and 2022 (N = 24,003)

Field	Overall (N = 24,003)		DDD (N = 23,689)		Non-DDD (N = 314)		p-value
	N	%^a^	N	%^a^	N	%^a^	
*Sociodemographic characteristics of children*							
Children							
Age (years)							
0	1453	5.34	1428	5.32	25	6.62	0.8563
1	3735	15.21	3690	15.23	45	13.92	
2	5374	22.99	5305	22.99	69	23.13	
3	5678	23.97	5606	23.97	72	23.92	
4	6063	25.44	5981	25.43	82	26.71	
5	1700	7.05	1679	7.07	21	5.69	
Sex							
Female	11,638	48.4	11,481	48.39	157	49.14	0.8153
Male	12,365	51.6	12,208	51.61	157	50.86	
*Sociodemographic characteristics of primary caregiver*							
Sex							
Female	19,921	81.76	19,648	81.69	273	87.01	0.0280
Male	4082	18.24	4041	18.31	41	12.99	
Age (years)							
Under 29	11,327	4834	11,149	48.21	178	58.96	0.0027
30–39	8921	35.98	8826	36.06	95	29.47	
40 or above	3755	15.69	3714	15.74	41	11.57	
Educational attainment							
None/Informal or religious education	11,417	52.59	11,297	52.69	120	44.61	0.0000
Primary	3941	16.25	3903	16.32	38	10.53	
Secondary	6709	24.37	6603	24.29	106	30.85	
Post-secondary	1936	6.78	1886	6.7	50	14.02	
Occupation							
Not employed/Unemployed	8041	35.78	7951	35.8	90	34.07	0.6593
Agriculture	6156	25.23	6090	25.25	66	23.21	
Unskilled and skilled manual work	3127	12.13	3071	12.11	56	13.66	
Sales/service/professional	6679	26.87	6577	26.85	102	29.06	
Partnership status							
Married/partnered	22,839	95.81	22,550	95.83	289	93.49	0.0448
Non-partnered	1164	4.19	1139	4.17	25	6.51	
*Sociodemographic characteristics of household head*							
Age (years)							
Under 29	3977	17.27	3907	17.20	70	22.87	0.0048
30–39	8522	35.35	8400	35.30	122	39.55	
40 or above	11,504	47.38	11,382	47.50	122	37.58	
Sex							
Female	5415	22.64	5329	22.6	86	25.8	0.2308
Male	18,587	77.36	18,359	77.4	228	74.2	
Educational attainment							
None/Informal or religious education	9565	44.20	9451	44.22	114	42.9	0.0100
Primary	2790	10.88	2765	10.93	25	7.23	
Secondary	7403	28.69	7306	28.71	97	26.54	
Post-secondary	4244	16.23	4166	16.14	78	23.34	
Occupation							
Not employed/Unemployed	3926	17.07	3900	17.17	26	9.43	0.0004
Agriculture	9604	41.67	9498	41.72	106	37.47	
Unskilled and skilled manual work	4150	15.70	4073	15.64	77	20.96	
Sales/service/professional	6322	25.55	6217	25.47	105	32.14	
Religion							
Islam	17,803	82.55	17,602	82.66	201	73.7	0.0000
Other	6200	17.45	6087	17.34	113	26.3	
Born state (Was the head of household born in the state)							
Yes	21,935	93.23	21,689	93.38	246	81.2	0.0000
No	2067	6.77	1999	6.62	68	18.8	
*Sociodemographic characteristics of households*							
Household residence status (since 1st July)							
Yes	23,823	99.41	23,519	99.43	304	97.24	0.0000
No	179	0.59	169	0.57	10	2.76	
Household nomad (moving cyclically or periodically at least one time per year)							
Yes	7258	32.59	7188	32.69	70	24.00	0.0040
No	16,744	67.41	16,500	67.31	244	76.00	
Wealth index							
Low	8117	36.82	8028	36.84	89	34.58	0.0000
Middle	7963	34.05	7885	34.18	78	23.82	
High	7923	29.13	7776	28.98	147	41.6	
*Malaria prevention practices*							
Household spray							
Yes	8937	39.09	8867	39.28	70	23.58	0.0000
No	15,066	60.91	14,822	60.72	244	76.42	
Ownership of mosquito nets							
Yes	17,630	76.24	17,447	76.37	183	65.18	0.0000
No	6373	23.76	6242	23.63	131	34.82	
*Caregiver information sources and knowledge toward SMC campaign*							
Heard about the date of SMC distribution in the one-month period prior to the final cycle							
Yes	18,283	79.25	18,171	79.79	112	35.55	0.0000
No	5720	20.75	5518	20.21	202	64.45	
Heard about SMC from local leader							
Yes	6050	29.19	6016	29.40	34	12.09	0.0000
No	17,953	70.81	17,763	70.60	280	87.91	
Heard about SMC from religious leader							
Yes	3918	15.90	3877	15.92	41	14.05	0.4313
No	20,085	84.10	19,812	84.08	273	85.95	
Heard about SMC from health facility staff							
Yes	5535	22.91	5474	22.93	61	21.76	0.6727
No	18,468	77.09	18,215	77.07	253	78.24	
Heard about SMC from CHW or SMC distributor							
Yes	5857	22.42	5787	22.49	70	16.84	0.0192
No	18,146	77.58	17,902	77.51	244	83.16	
Heard about SMC from radio							
Yes	3564	16.01	3538	16.11	26	8.04	0.0003
No	20,439	83.99	20.151	83.89	288	91.96	
Heard about SMC from town announcer							
Yes	9365	42.08	9295	42.21	70	31.29	0.0015
No	14,638	57.92	14,394	57.79	244	68.71	
Heard about SMC from word of mouth (e.g. friends or family)							
Yes	2134	8.90	2110	8.93	24	6.29	0.1376
No	21,869	91.10	21,579	91.07	290	93.71	
Knowledge of SMC purpose							
Yes	19,782	85.16	19,603	85.46	179	60.54	0.0000
No	4221	14.84	4086	14.54	135	39.46	
Awareness of SMC AQ importance							
Yes	19,898	85.65	19,725	85.98	173	58.19	0.0000
No	4105	14.35	3964	14.02	141	41.81	
Awareness of SMC adverse reactions							
Yes	17,204	74.33	17,056	74.61	148	51.67	0.0004
No	6799	25.67	6633	25.39	166	48.33	
Knowledge of SMC eligibility							
Yes	19,586	84.83	19,413	85.16	173	58.26	0.0000
No	4417	15.17	4276	14.84	141	41.74	
Knowledge of age protection							
Yes	18,123	79.26	17,970	79.57	153	53.92	0.0000
No	5880	20.74	5719	20.43	161	46.08	
Belief in SMC effectiveness							
Yes	20,365	87.05	20,164	87.32	201	65.51	0.0000
No	3639	12.95	3525	12.68	113	34.49	

^a^Weighted proportion based on corrected χ^2^ test. *SMC* seasonal malaria chemoprevention, *DDD* door-to-door distribution, *AQ* Amodiaquine

This study also aimed to understand whether there were differences in adherence to complete administration of SMC medicines and caregiver reporting of adverse reactions between those who accessed SMC through DDD and non-DDD. Adherence to administration of both Day 2 and Day 3 AQ and its association with access to SMC medicines by non-DDD were examined (Table 2). Variables related to caregiver reporting of adverse reactions to SMC medicine, including the occurrence of adverse reactions to SMC medicines, the types of adverse reactions, caregiver actions in the event of adverse reactions were also included in the analysis (Table 2).

**Table 2:** Differences between access to SMC medicines in caregiver self-reported adherence to SMC medicines and caregiver actions in the event of adverse reactions to SMC medicines

	Category	Sample	Total		DDD		Non-DDD		F-statistic	p-value
			n	%^a^	n	%^a^	n	%^a^		
*Adherence to administration of both Day 2 and Day 3 AQ*										
Received day 2 + 3 AQ	Yes	24,003	23,582	98.36	23,313	98.50	269	86.74	237.226	0.000
	No		4212	1.64	376	1.50	45	13.26		
*Occurrence of adverse reactions to SMC medicines*										
Self-reported adverse reactions	Yes	24,003	3827	16.03	3774	16.01	53	17.89	0.617	0.432
	No		20,176	83.97	19,915	83.99	261	82.11		
*Types of adverse reactions to SMC medicines*										
Severe vomiting	Yes	3827	1236	32.78	1224	32.91	12	23.24	1.972	0.160
	No		2591	67.22	2550	67.09	41	76.76		
Diarrhoea	Yes	3827	299	7.99	296	8.030	3	5.46	0.460	0.498
	No		3528	92.01	3478	91.97	50	94.54		
Skin reaction or itch	Yes	3827	273	6.94	270	6.940	3	6.51	0.012	0.914
	No		3554	93.06	3504	93.06	50	93.49		
Yellow eyes	Yes	3827	293	7.46	275	6.99	18	41.74	69.443	0.000
	No		3534	92.54	3499	93.01	35	58.26		
Sleeplessness	Yes	3827	338	8.19	336	8.24	2	4.12	1.077	0.300
	No		3489	91.81	3438	91.76	51	95.88		
Fever	Yes	3827	1442	39.61	1422	39.71	20	32.53	0.951	0.330
	No		2385	60.39	2352	60.29	33	67.47		
Loss of appetite	Yes	3827	425	11.6	423	11.73	2	1.83	8.237	0.004
	No		3402	88.4	3351	88.27	51	98.17		
Other responses	Yes	3827	296	5.55	291	5.55	5	5.34	0.006	0.936
	No		3531	94.45	3483	94.45	48	94.66		
*Caregiver actions in the event of adverse reactions to SMC medicines*										
Report to SMC distributor or health facility	Yes	3827	2866	78.41	2833	78.64	33	61.63	6.983	0.008
	No		961	21.59	941	21.36	20	38.37		
Reasons for non-reporting	Do not know	961	337	38.09	326	37.34	11	68.68	3.379	0.019
	Too far or limited access		73	8.25	70	8.21	3	9.80		
	Consider the reaction mild		494	48.47	489	49.22	5	17.67		
	Others		57	5.2	56	5.23	1	3.85		

^a^Weighted proportion based on corrected χ^2^ test

*SMC* seasonal malaria chemoprevention, *DDD* door-to-door distribution, *AQ* Amodiaquine

Definitions of all variables of interest and corresponding survey questions can be found in Additional file 1: Table S1.

### Statistical analysis

First, this study estimated the coverage of children receiving SMC medicines through non-DDD and described the characteristics of the study sample. Frequency (n) and weighted proportion (%) were examined. Survey weights were calculated based on the inverse of the probability of selection for each observation using the population size of each state and the sample of survey respondents to account for the disproportionate representation of certain states. Differences in baseline characteristics between DDD and non-DDD groups were compared using Pearson chi-squared (χ^2^) tests using second-order corrections converting results into a F-statistics Rao and Scott [23].

Additionally, this study described the distribution of non-DDD sources of SMC medicines w. These included family or friends, health facility staff, fixed-point distribution by SMC distributors, unofficial fixed-point distribution, private purchase, distribution by SMC distributors in another location, and any other source (see Additional file 1: Table S2 for full category definitions).

Second, multilevel logistic regression models were performed to identify predictors of access to SMC medicines through non-DDD. The regression model included random intercepts for survey year, nested within the local government area (LGA) and state. Variables were selected using a forward stepwise approach. In brief, starting with an empty model, variables were sequentially added based on Wald test results, and they were retained if they improved model fit based on a significance level (p-value < 0.05) [24]. Unadjusted univariate logistic regression was also performed for all variables as a reference. Results were reported as odds ratios (ORs) with 95% confidence intervals (95% CI).

Finally, the association between access to SMC medicines and caregiver reporting of adherence to SMC medicines, as well as the association between access to SMC medicines and caregiver actions in the event of adverse reactions to SMC medicines, were assessed using weighted proportions and corrected χ^2^ tests. Non-DDD access were further subdivided into non-DDD access via CDs or health facility personnel and informal non-DDD access (e.g., private purchase), and compared their differences in both caregiver reporting of adherence to SMC medicines and caregiver actions in the event of adverse reactions to SMC medicines as a reference, using weighted proportions and corrected χ^2^ tests.

Data were analysed using Stata 17.0, and post-sampling weights at the state level were applied throughout the descriptive and statistical analyses.

### Ethical considerations

EoR surveys in Nigeria were developed in collaboration with, and approved by, the Nigerian National Malaria Elimination Programme and the National Ministry of Health.

## Results

### Proportion of children receiving SMC medicines through non-DDD

The final analytic sample included data from 24,003 caregivers of eligible children who received Day 1 SPAQ in the final cycle of SMC between 2021 and 2022 from Nigeria (Fig. 3). The proportion of children receiving SMC medicines but were not visited by CDs (non-DDD access to SMC medicines) was 1.0% (238 of 24,003). In addition, there was 0.3% (76 of 23,765) of children received medicines outside but not at the time of household visit by CDs (non-DDD access to SMC medicines). Therefore, the overall proportion of eligible children who accessed SMC medicines through non-DDD was 1.3% (314 of 24,003). In the Federal Capital Territory (FCT), however, there was 14.2% (232 of 1635) eligible children did not receive SMC medicines, and 2.6% (37 of 1403) of those recipients accessed medicines through non-DDD (Additional file 1: Table S3).

### Sociodemographic characteristics of children, caregivers, and heads of household, households receiving SMC medicines through non-DDD

Table 1 presents the sociodemographic characteristics of children, caregivers, and households accessing SMC medicines through DDD and non-DDD. Caregivers in the non-DDD group were more likely to be female, younger, with higher education level, and non-partnered. Heads of household accessing SMC via non-DDD were more likely to be younger, highly educated, engaged in sales/service/professional work, Muslim, and born outside of the state of current residence. Households in the non-DDD group were more likely to reside in the implementing areas after annual SMC initiation, experience cyclic or periodic migration at least once a year, and have higher wealth. Furthermore, non-DDD was associated with ownership of mosquito nets and indoor residual spray. Caregivers accessing SMC via non-DDD sources were more likely to have heard the date of SMC distribution in the one-month period prior to the final cycle, and to have ever heard about SMC from local leaders, radio, and town announcers. However, caregivers accessing SMC via non-DDD were less likely to have knowledge of the purpose, age eligibility, reason for the eligible age range for SMC, awareness of the importance of AQ and adverse reactions, and belief in the effectiveness of SMC.

### Distribution of channels to non-DDD access to SMC medicines

More than one-third (39.5%, 120 of 314) and one-fifth (25.4%, 79 of 314) of channels to non-DDD access to SMC medicines were via health facility staff, and CDs in another location, respectively (Fig. 4). Family and friends accounted for 15.5% (52 of 314) of access to SMC medicines through non-DDD. Other channels to obtain SMC medicines included fixed point distribution by CDs (9.7%, 30 of 314), unofficial fixed-point distribution (1.7%, 7 of 314), private purchase (3.6%, 8 of 314), and others (4.6%, 18 of 314) (see Additional file 1: Table S1 for detailed definitions).

### Factors predicting access to SMC medicines

**Fig. 5:**
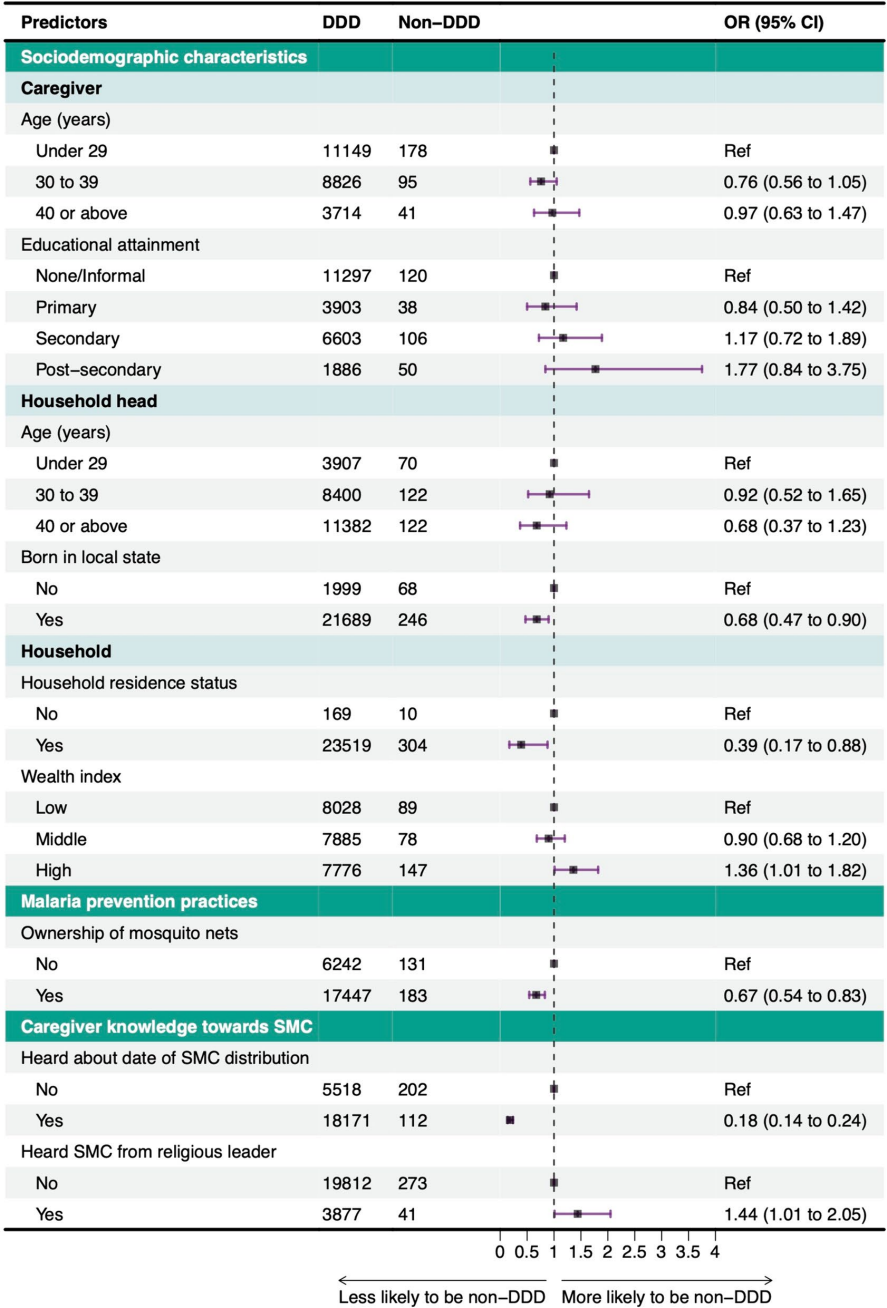
Multivariate logistic regression results of factors associated with access to SMC medicines (N = 24,003). The reference line at 1 indicates no increase or decrease in the likelihood of access to SMC outside household visits. The 95% confidence intervals (CIs) also are plotted. *SMC* seasonal malaria chemoprevention, *DDD* door-to-door distribution, *Ref* Reference category, *OR (adjusted)* Odds ratio

Figure 5 presents the results of the multiple logistic regression model after forward stepwise selection. After mutual covariate adjustment, odds of access to SMC medicines through non-DDD were lower among children in households where heads of household were born in the local state than those with heads of household born outside of the state (OR = 0.68, 95% CI 0.47 to 0.90). Similarly, children in households residing in the same state since the first cycle of the SMC round had lower odds of accessing SMC medicines through non-DDD (OR = 0.39, 95% CI 0.17 to 0.88). Compared with households with low wealth index, those categorized as having high wealth index had higher odds of accessing SMC medicines through non-DDD (OR = 1.36, 95% CI 1.01 to 1.82). Households that owned mosquito nets had lower odds of accessing SMC through non-DDD than households that did not have mosquito nets (OR = 0.67, 95%CI 0.54 to 0.83). Caregivers that heard the date of SMC distribution within the last month (i.e., one-month period prior to the final SMC cycle) had lower odds of accessing SMC medicines through non-DDD than caregivers that did not hear the date of SMC delivery date within the last month (OR = 0.18, 95%CI 0.14 to 0.24). Caregivers who ever heard of SMC from a religious leader had higher odds of accessing SMC through non-DDD (OR = 1.44, 95% CI 1.01–2.05). Results of univariate regressions are shown in Additional file 1: Fig. S1.

### Caregiver reporting of adherence to complete administration of SMC medicines and caregiver actions in the event of adverse reactions

Caregivers who accessed SMC medicines through non-DDD were less likely to adhere to administration of both Day 2 and Day 3 AQ doses than those accessing medicines through DDD (86.74% vs. 98.50%; F(1, 24,002) = 237.226, p < 0.001) (Table 2). The proportion of caregivers of eligible children receiving SMC medicines who reported adverse reactions was 16.03% (3,827 of 24,003). There was no significant difference in the occurrence of caregiver-reported adverse reactions between DDD and non-DDD (16.01% vs. 17.89%; F(1, 24,002) = 0.617, p = 0.432). However, caregivers who accessed SMC medicines through non-DDD were more likely to report their child exhibiting yellow eyes than those who obtained SMC medicines through DDD (41.74% vs. 6.99%; F(1, 24,002) = 69.443, p < 0.001). Loss of appetite was more common among children in household with access via DDD compared with non-DDD (11.73% vs. 1.83%; F(1, 24,002) = 8.237, p = 0.004).

There were differences in caregiver self-reported adverse reactions to SMC medicines among children between DDD and non-DDD groups. Caregivers that accessed SMC medicines through non-DDD were less likely to report adverse reactions to CDs or health facility personnel than caregivers that accessed SMC medicines through DDD (61.63% vs. 78.64%, F(1, 24,002) = 6.983, p = 0.008).

Table 2 shows differences in reasons for non-reporting of adverse reactions to SMC medicines in children. In households accessing SMC through non-DDD, the proportion of caregivers who did not report adverse events because they did not know they should report them was approximately twice as high compared with households accessing SMC through DDD (68.68% vs. 37.34%). Conversely, the proportion of caregivers who considered adverse reactions experienced by their children as mild was approximately two times lower among households accessing SMC through non-DDD than those accessing SMC through DDD (17.67% vs. 49.22%).

Results of differences between non-DDD access to SMC medicines in caregiver self-reported adherence to SMC medicines and caregiver actions in the event of adverse reactions to SMC medicines are shown in Additional file 1: Table S4. No statistically significant difference was found in caregiver reporting adherence to AQ administration (88.98% vs. 80.14%; F(1, 24,002) = 3.639, p = 0.057) and caregivers reporting to SMC distributors or health facility personnel in the event of children's adverse reactions (56.19% vs. 79.12%; F(1, 24,002) = 2.215, p = 0.143) between non-DDD via SMC distributors or health facility personnel, and informal non-DDD.

## Discussion

### Summary of key findings

Distribution of SMC medicines has predominantly relied on DDD, with a small (2%, 314 of 24,003) but non-negligible proportion of households accessing SMC medicines through non-DDD during the final cycle of SMC rounds in 2021 and 2022 in Nigeria. Over two-thirds of non-DDD instances were considered to be through legitimate channels, such as health facility staff and distribution by CDs in other locations; however, these do not adhere to current SMC delivery protocols.

This study characterized households administering SMC medicines to their eligible children through non-DDD during the final cycle of SMC rounds in 2021 and 2022. Three socioeconomic factors predicted households that accessed SMC medicines through non-DDD, including head of household’s place of birth, household residence status (living in the study state since the first cycle of the SMC round), and wealth index. Head of households from the outside-born population and households residing in this implementing location after the first cycle of SMC had a higher odds of accessing SMC medicines through non-DDD. It could be explained as households only hearing of dates of the upcoming SMC after the initiation of the annual round of SMC or happening to travel outside of the location during the distribution period of SMC. Similarly, a higher wealth index was positively associated with access to SMC medicines through non-DDD, which is expected as it is of financial affordability and may be more convenient for wealthier households to administer medicines to children ahead of the SMC cycle without waiting around all day for the CDs. The study also found that caregivers hearing about the date of SMC distribution in a month period prior to the final cycle was negatively associated with access to SMC medicines through non-DDD. Households informed of SMC delivery dates may be more likely to stay at home and wait for DDD.

Moreover, both complete adherence to administration of SMC medicines and caregiver responses to children’s adverse reactions to SMC medicines differed between DDD and non-DDD groups. Compared with households accessing SMC medicines through DDD, we found a significantly higher proportion of non-adherence to administration of both Day 2 and Day 3 AQ and a higher proportion of caregivers accessing SMC unaware that they should report children’s adverse reactions to health facilities or CDs in non-DDD group. These findings imply that access to SMC medicines through non-DDD might have reduced interpersonal interactions between family members and CDs from the local community, consistent with the caregiver’s perspective on DDD [16]. Moreover, no statistically significant difference between non-DDD access via CDs or health facility personnel, and informal non-DDD access, in both caregiver responses to adverse reactions and caregiver responses to children’s adverse reactions further emphasised that in this regard CDs or health facility personnel who deliver medicines in other places (just not at household-to-household door) cannot significantly contribute to positive outcomes in these aspects as DDD by CDs (Additional file 1: Table S4).

### Implications of study findings

The latest WHO guidelines on SMC provide implementers greater flexibility in SMC distribution channels; SMC programmes could employ DDD in rural areas with lower population density, mobile outreach teams for nomadic populations, and fixed-point delivery in urban settings or schools [6, 7]. The study findings highlight potential downsides that may arise when transitioning from large-scale DDD to non-DDD in terms of adherence to SMC medicines and caregiver actions to adverse reactions. It is essential to develop mitigation strategies to ensure the success of alternative delivery approaches if adopted, especially to ensure high adherence to administration of full courses of SMC medicines. For example, during school-based SMC delivery, education about malaria infection and medication supervision by schoolteachers or administrators may play a crucial role in mitigating non-DDD-induced risks and maintaining high coverage in schools efficiently [25–27]. Nevertheless, it's important to note that not all school-aged children attend primary school regularly, entailing the need for auxiliary delivery approaches.

It is also vital to enhance communication strategies, such as via community leaders, radio, and printed materials, to inform caregivers about upcoming SMC cycles and distribution locations, regardless of different delivery approaches employed. Furthermore, lead mothers visit intervention, female residents aged 18 years and above who visit caregivers door-to-door on Day 2 following Day 1 DDD by SMC distributors to disseminate SMC messages and remind AQ administration, play a promising role in building a strong connection with caregivers to adopt healthy behaviours and malaria prevention in Nigeria [28, 29]. Households visited by lead mothers during SMC had higher odds of accessing SMC medicines via DDD using data from 2022 EoR survey, demonstrating the role of community peer-support and motivation systems in fostering DDD and complementing non-DDD. Moreover, ongoing pharmacovigilance of SMC medicines is required to build an evaluation of the benefit-risk of deployment of different distribution channels and novel chemoprevention medicine combinations.

### Study strengths and limitations

Previous analysis of access to SMC medicines through non-DDD has been limited to basic summary statistics from annual coverage reports and anecdotes [8]. This is the first quantitative assessment to date to explore potential predictors of access to SMC medicines through non-DDD and describe its association with adherence to administration of SMC medicines and adverse reaction reporting. It can serve as an indicator for evaluating compliance with SMC delivery protocols. EoR surveys provided a rich data source and large sample size to address new research questions without the need for dedicated surveys. A significant strength of the survey was that it was conducted by external investigators not affiliated with the SMC programme, which can enhance objectivity and reduce investigator bias. Besides, the large representative dataset (n = 25,278) covering multiple states ensures generalizability of the results across SMC-eligible areas in Nigeria.

As with most of secondary survey data analyses, this study may have been subject to recall bias and potential social-desirability bias due to its reliance on self-reporting responses by caregivers. Recall bias may have been further increased due to the time lag of up to two months between the final SMC cycle and EoR surveys in some areas. Furthermore, there is a possibility of misreporting and misclassification of access to SMC medicines, as caregivers may not accurately recall whether the medicines their child received were specifically for SMC, malaria treatment, or pain relief. This could introduce biases in the estimates of predictors and questions related to adherence and adverse reactions. However, the logic framework of the survey design, including logic and conditional questions, may have helped mitigate these biases by assisting respondents in recalling survey information. Another minor limitation of this study is the use of a three-quintile wealth index based on older indicators from the 2012/13 General Household Panel Survey and the Simple Poverty Scorecard™ Nigeria [30].

### Future research directions

Future research could focus on comparing the outcomes of various medicine distribution approaches on SMC’s coverage and effectiveness in preventing malaria before and after deployment of non-DDD in pilot areas and examining pharmacovigilance (e.g., adverse reactions) associated with these new distribution approaches. EoR surveys can continue to represent a useful monitoring tool to summarize the distribution of access to SMC medicines by different channels and identify potential implementation challenges. It allows implementers to identify and investigate specific communities and villages where survey data demonstrate a notable increase in the frequency of access to SMC medicines outside of the approach prescribed by local implementation protocols, which can inform potential actions for CD training. Besides, relevant survey questions could also be added to existing routine coverage surveys to address new research questions, such as the implementation of medication distribution to nomadic populations or the association between travel time or distance to SMC medicine fixed-point distribution sites in pilot areas, and outcomes of malaria prevention (e.g., caregivers’ knowledge of SMC or adherence to SMC medicines).

## Conclusion

Overall, this study profiled characteristics of primary caregiver, child, and household accessing SMC medicines through non-DDD and demonstrated the potential negative outcomes of non-DDD on caregiver adherence to AQ administration and children’s adverse reaction reporting in Nigeria. Findings point to the need to adopt mitigation strategies should SMC delivery protocols be adapted to include non-DDD channels.

### Supplementary Information

**Additional file 1: Table S1.** Operational definitions of study variables. **Table S2.** Operational definitions of channels to non-DDD access to SMC medicines. **Table S3.** Coverage of Day 1 SPAQ by region (state) in Nigeria. **Table S4.** Differences between non-DDD access to SMC medicines in caregiver self-reported adherence to SMC medicines and caregiver actions in the event of adverse reactions to SMC medicines. **Fig. 1.** Univariate logistic regression results.

## Data Availability

Processed data supporting the findings of this study are included in this published article and its supplementary information files. Original datasets generated and analysed during the current study are available from the corresponding author on reasonable request.

## Abbreviations

WHO: World Health Organization
SMC: Seasonal malaria chemoprevention
SP: Sulfadoxine-pyrimethamine
AQ: Amodiaquine
CD: Community distributor
DDD: Door-to-door distribution
FCT: Federal Capital Territory
EoR: End-of-round
LGA: Local government area
