# Supplementary material for: Predictors of accessing seasonal malaria chemoprevention medicines through non-door-to-door distribution in Nigeria
Source: Malar J. 2024 May 3;23:131. doi: 10.1186/s12936-024-04964-5 (PMC11067122; doi:10.1186/s12936-024-04964-5)
Supplement: Supplementary file 1 — Additional file 1: Table S1. Operational definitions of study variables. Table S2. Operational definitions of channels to non-DDD access to SMC medicines. Table S3. Coverage of Day 1 SPAQ by region (state) in Nigeria. Table S4. Differences between non-DDD access to SMC medicines in caregiver self-reported adherence to SMC medicines and caregiver actions in the event of adverse reactions to SMC medicines. Fig. 1. Univariate logistic regression results. [file 12936_2024_4964_MOESM1_ESM.docx]

# Additional materials

***Table S1***. Operational definitions of study variables

| Field | Question | Response |
| --- | --- | --- |
| Sociodemographic characteristics of children | | |
| Age | What is the [selected child]’s age in years? | 0 (Less than one year)12345 |
| Sex | What is the [selected child]’s gender? | FemaleMale |
| Sociodemographic characteristics of the primary caregiver | | |
| Age | What is the age of [selected child]’s primary caregiver? | Under 29 years30-3940 or more years |
| Sex | What is the gender of [selected child]’s primary caregiver? | FemaleMale |
| Partnership status | What is the primary caregiver’s partnership status? | Married/PartneredUnmarried/Unpartnered |
| Educational attainment | What is the highest level of education that the caregiver has completed? | None or informalPrimarySecondaryPost-secondary |
| Occupation | What is the caregiver’s occupation? | None or UnemployedAgricultureManual workSales/Services/Professional |
| Sociodemographic characteristics of the head of household | | |
| Age | What is the head of household’s age? | Under 29 years30-3940 or more years |
| Sex | What is the head of household’s gender? | FemaleMale |
| Educational attainment | What is the highest level of education that the head of household has completed? | None or informalPrimarySecondaryPost-secondary |
| Occupation | What is the head of household occupation? | None or UnemployedAgricultureManual workSales/Services/Professional |
| Religion | What is the religion of the head of household? | IslamOther |
| Born state | Was the head of household born in this state? | YesNo |
| Sociodemographic characteristics of household | | |
| Residence status | Has this household been in this location since 1st July (2021/2022)? | YesNo |
| Seasonal migration | Does this household not living continually in this location, but moving cyclically or periodically at least one time per year? | YesNo |
| Wealth index^a^ | What is the household’s wealth index? | LowMiddleHigh |
| Malaria prevention practices | | |
| Indoor residual spray | Did the structure which you live in receive indoor residual spray during the last 12 months? | YesNo |
| Mosquito nets | Do you or your household own a mosquito net? | YesNo |
| Caregiver information sources and knowledge toward SMC | | |
| Heard about SMC within last month (one month prior to the final cycle) | Did you hear information within the last month about the date that SMC would take place? | YesNo |
| Heard SMC from local leader | Have you ever heard about "SMC" or "Seasonal Malaria Chemoprevention" from local leader? | YesNo |
| Heard SMC from religious leader | Have you ever heard about "SMC" or "Seasonal Malaria Chemoprevention" from religious leader? | YesNo |
| Heard SMC from health facility staff | Have you ever heard about "SMC" or "Seasonal Malaria Chemoprevention" from health facility staff? | YesNo |
| Heard SMC from community distributor | Have you ever heard about "SMC" or "Seasonal Malaria Chemoprevention" from community distributor? | YesNo |
| Heard SMC from radio | Have you ever heard about "SMC" or "Seasonal Malaria Chemoprevention" from radio? | YesNo |
| Heard SMC from town announcer | Have you ever heard about "SMC" or "Seasonal Malaria Chemoprevention" from town announcer? | YesNo |
| Heard SMC from word of mouth | Have you ever heard about "SMC" or "Seasonal Malaria Chemoprevention" from word of mouth (e.g., friend)? | YesNo |
| SMC purpose | Do you know what the purpose of SMC is?If the caregiver mentions protecting children against malaria, please select “yes”. If the caregiver gives no answer, or an incorrect answer, please select “no”. | YesNo |
| SMC eligibility | Do you know which children are eligible to receive SMC?Select yes if the caregiver mentions children under five years. | YesNo |
| SMC age protection | Do you know why it is important that only children aged under five years receive SMC?If the caregiver mentions that SMC does not offer sufficient protection for older children, or that older children are less at risk of malaria and therefore do not need SMC, please select “yes”. If the caregiver does not give a response or gives an incorrect response, please select “no”. | YesNo |
| SMC AQ importance | When SMC is administered to eligible children, the SMC medicine distributor leaves two drug doses behind for caregivers to give to their children daily at home over the next two days. Do you known why it is important to give children these doses?If the caregiver mentions that it is important for children to receive all doses to ensure full protection against malaria, please select “yes”. If the caregiver does not give a response or gives an incorrect response, please select “no”. | YesNo |
| SMC adverse events | Do you know what you should do if a child experiences an adverse reaction after taking SMC medicines given to them by a drug distributor?If the caregiver mentions that the child should be taken to a clinic or that it should be reported to a SMC drug distributor (or similar answer), please select “yes”. If the caregiver gives no answer, or an incorrect answer, please select “no”. | YesNo |
| Brief in SMC effectiveness | Do you believe that SMC drugs are effective at protecting young children from malaria during the rainy season? | YesNo |
| Caregiver reporting of adherence to SMC medicines and caregiver actions in the event of adverse reactions to SMC medicines | | |
| Adherence to Day 2 & Day 3 | Did you administer the second SMC dose to [selected child] on both the Day 2 and Day 3? | YesNo |
| Occurrence of adverse reactions | Did [selected child] experienced any reaction to either SP on Day 1 or AQ on Day 2 or Day 3? | YesNo |
| Types of adverse reactions ^b^ | What type of adverse reactions did [selected child] experience? | Severe vomitingDiarrhoeaSkin reaction or itchYellow eyesSleeplessnessFeverLoss of appetiteOther responses |
| Caregiver reporting of adverse reactions | Did you tell the SMC distributor or staff at a health facility about this adverse reaction? | YesNo |
| Reasons for non-reporting of adverse reactions | Why didn’t you tell distributor/health facility about this adverse reaction? | Didn’t know this was an optionToo far or limited physical accessDidn’t consider the reaction serious enoughOther responses |

^a^ Household wealth index was determined by the Simple Poverty Scorecard™ Nigeria (Schreiner, 2015) which uses ten poverty indicators to estimate the likelihood of a household being in a poverty.^30^ All ten indicators were available in the EOR surveys and used in this analysis, including (1) number of household members, (2) number of bedrooms, (3) type of the dwelling structure, (4) type of toilet facility, (5) ownership of cooking utensils, (6) number of mattresses, (7) TV ownership, (8) number of mobile phones, (9) motor vehicle ownership, and (10) presence of agricultural activities. Each question contains points for different responses. We summed up the points from the ten questions to create an overall score for each household. Wealth index was assigned as lowest tertile, middle tertile, or highest tertile based on the overall score distribution.

# ^b^ Types of adverse reactions to SMC medicines among children were not non-exclusive categories whereas other variables’ responses were exclusive categories.

## Table S2. Operational definitions of channels to non-DDD access to SMC medicines

| Channel | Definition | SMC community distributor^a^ | SMC programme^b^ |
| --- | --- | --- | --- |
| Family or friend | Caregivers obtained the SMC medicines from family members or friends that kept in stock | No | No |
| Health facility staff | Caregivers obtained the SMC medicines from health facility staff when they visited the clinics or outreach clinics for any reasons | No | Yes |
| Fixed-point distribution by SMC distributors | SMC medicines were distributed by SMC distributors from one pre-agreed location, and caregivers travelled there to collect SMC medicines | Yes | Yes |
| Unofficial fixed-point distribution | SMC medicines were distributed by a person not affiliated with the SMC programme, and caregivers travelled there to collect SMC drugs (e.g., drugs given out freely in a market) | No | No |
| Private purchase | Caregivers bought the same SMC medicines distributed by the SMC programme with their own money | No | No |
| Distribution by SMC distributors in another location | SMC medicines were distributed to a caregiver in the street or at their workplace by SMC distributors, not the caregiver’s home | Yes | Yes |
| Other sources^c^ | Any other sources not included | No | No |

SMC, Seasonal malaria chemoprevention

^a^ Whether SMC medicines are distributed by SMC community distributor in the channel.

^b^ Whether the channel is a recognised as an official channel by SMC programme.

^c^ This category may have included other sources for receiving medicines through non-door-to-door delivery.

## Table S3. Coverage of Day 1 SPAQ by region (state) in Nigeria

| **Region** | **SMC Coverage** | | | | | | **Channel of access to SMC medicines^a^** | | | |
| --- | --- | --- | --- | --- | --- | --- | --- | --- | --- | --- |
|  | **Total** | | **Received** | | **Did not receive** | | **DDD** | | **Non-DDD** | |
|  | n | % | n | % | n | % | n | % | n | % |
| Bauchi | 2677 | 15.77 | 2611 | 97.88 | 66 | 2.12 | 2588 | 99.22 | 23 | 0.78 |
| Borno | 2863 | 17.43 | 2754 | 96.26 | 109 | 3.74 | 2714 | 98.47 | 40 | 1.53 |
| FCT | 1635 | 5.01 | 1403 | 85.81 | 232 | 14.19 | 1366 | 97.36 | 37 | 2.64 |
| Jigawa | 1483 | 6.49 | 1440 | 97.10 | 43 | 2.90 | 1429 | 99.24 | 11 | 0.76 |
| Kano | 1340 | 11.85 | 1269 | 94.70 | 71 | 5.30 | 1254 | 98.82 | 15 | 1.18 |
| Kebbi | 2795 | 10.72 | 2762 | 98.78 | 33 | 1.22 | 2740 | 99.18 | 22 | 0.82 |
| Kogi | 2636 | 6.47 | 2466 | 92.22 | 170 | 7.78 | 2409 | 97.91 | 57 | 2.09 |
| Nasarawa | 2699 | 7.38 | 2583 | 95.67 | 116 | 4.33 | 2549 | 98.69 | 34 | 1.31 |
| Oyo | 1358 | 1.35 | 1280 | 94.26 | 78 | 5.74 | 1266 | 98.91 | 14 | 1.09 |
| Plateau | 2678 | 7.11 | 2595 | 97.09 | 83 | 2.91 | 2552 | 98.48 | 43 | 1.52 |
| Sokoto | 2979 | 10.42 | 2840 | 95.12 | 139 | 4.88 | 2822 | 99.33 | 18 | 0.67 |

FCT, Federal Capital Territory; DDD, door-to-door distribution

^a^ Access to SMC medicines is conditional on households that received SMC medicines. For example, the sample of access to SMC medicines in Bauchi was comprised of the sample of households that received medicines (N = 2611).

## Table S4. Differences between non-DDD access to SMC medicines in caregiver self-reported adherence to SMC medicines and caregiver actions in the event of adverse reactions to SMC medicines

|  | **Category** | **Sample** | **Total** | | **non-DDD via SMC distributors or health facility personnel^b^** | | **Informal non-DDD^c^** | | **F-statistic** | **p-value** |
| --- | --- | --- | --- | --- | --- | --- | --- | --- | --- | --- |
|  |  |  | n | %^a^ | n | %^a^ | n | %^a^ |  |  |
| **Adherence to administration of both Day 2 and Day 3 AQ** | | | | | | | | | | |
| Received day 2+3 AQ | Yes | 314 | 269 | 86.74 | 201 | 88.98 | 68 | 80.14 | 3.639 | 0.057 |
|  | No |  | 45 | 13.26 | 28 | 11.02 | 17 | 19.86 |  |  |
| **Occurrence of adverse reactions to SMC medicines** | | | | | | | | | | |
| Self-reported adverse reactions | Yes | 314 | 53 | 17.89 | 38 | 18.28 | 15 | 16.74 | 0.080 | 0.778 |
|  | No |  | 261 | 82.11 | 191 | 81.72 | 70 | 83.26 |  |  |
| **Caregiver actions in the event of adverse reactions to SMC medicines** | | | | | | | | | | |
| Report to SMC distributor or health facility | Yes | 53 | 33 | 61.63 | 23 | 56.19 | 10 | 79.12 | 2.215 | 0.143 |
|  | No |  | 20 | 38.37 | 15 | 43.81 | 5 | 20.88 |  |  |
| Reasons for non-reporting | Do not know | 20 | 11 | 68.68 | 9 | 75.76 | 2 | 20.96 | 5.445^d^ | 0.045^d^ |
|  | Too far or limited access |  | 3 | 9.80 | 2 | 9.70 | 1 | 10.48 |  |  |
|  | Consider the reaction mild |  | 5 | 17.67 | 3 | 10.13 | 2 | 68.55 |  |  |
|  | Others |  | 1 | 3.85 | 1 | 4.42 | 0 | 0.00 |  |  |

^a^ Weighted proportion based on corrected χ2 test. Abbreviations: SMC, seasonal malaria chemoprevention; DDD, door-to-door distribution; AQ, Amodiaquine.

^b^ Non-DDD via SMC distributors or health facility personnel included health facility personnel (N = 120), fixed-point distribution by SMC distributors (N = 30), and distribution by SMC distributors in another location (N = 79). see Table S1 for detailed definitions.

^c^ Informal non-DDD included unofficial fixed-point distribution (N = 7), private purchase (N = 8), family or friends (N = 52), others (N = 18). See Table S1 for detailed definitions.

^d^ F-statistic and p-values were generated by corrected χ2 test as other pairs of variables. However, due to the quite small sample size of reasons for non-reporting (which may violate assumption of χ2 test), we also used Fisher’ exact test to assess the association between reasons for non-reporting and non-DDD access (non-DDD via SMC distributors or health facility personnel vs. informal non-DDD), which demonstrated insignificant association with a p-value of 0.840.

## Fig. 1 Univariate logistic regression results


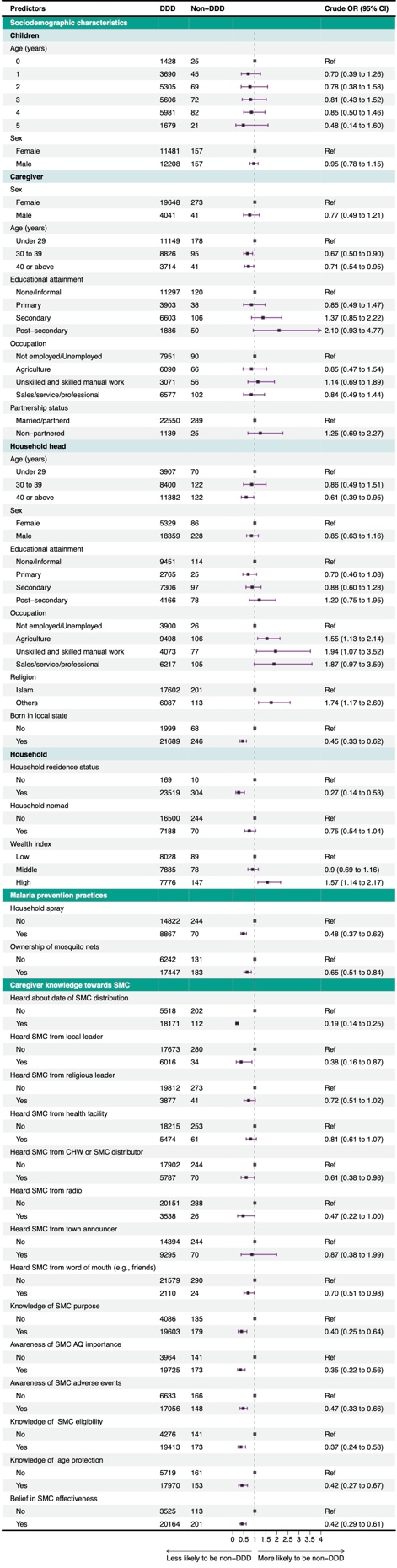


The reference line at 1 indicates no increase or decrease in the likelihood of access to SMC outside household visits. The 95% confidence intervals (CIs) also are plotted. Abbreviations: SMC, Seasonal malaria chemoprevention; DDD, door-to-door distribution; Ref, Reference category; OR, (adjusted) Odds ratio.
